# Mobip: a lightweight model for driving perception using MobileNet

**DOI:** 10.3389/fnbot.2023.1291875

**Published:** 2023-12-04

**Authors:** Minghui Ye, Jinhua Zhang

**Affiliations:** School of Mechanical and Electrical Engineering, Guangzhou University, Guangzhou, China

**Keywords:** self-driving, multi-task learning, semantic segmentation, traffic object detection, lightweight network

## Abstract

The visual perception model is critical to autonomous driving systems. It provides the information necessary for self-driving cars to make decisions in traffic scenes. We propose a lightweight multi-task network (Mobip) to simultaneously perform traffic object detection, drivable area segmentation, and lane line detection. The network consists of a shared encoder for feature extraction and two decoders for handling detection and segmentation tasks collectively. By using MobileNetV2 as the backbone and an extremely efficient multi-task architecture to implement the perception model, our network has great advantages in inference speed. The performance of the multi-task network is verified on a challenging public Berkeley Deep Drive(BDD100K) dataset. The model achieves an inference speed of 58 FPS on NVIDIA Tesla V100 while still maintaining competitive performance on all three tasks compared to other multi-task networks. Besides, the effectiveness and efficiency of the multi-task architecture are verified via ablative studies.

## 1 Introduction

Cameras have been widely used to construct the perception system of self-driving cars in recent years, bringing the demand for intelligent visual perception algorithms to process the captured images. Visual perception systems on autonomous driving cars can process images of the surrounding environment and subsequently provide vehicles with information about the traffic scenes, which builds the basics for trajectory planning and decision-making. Specifically, the images captured by cameras can be used to detect the position and size of traffic objects such as cars, pedestrians, and obstacles. In addition, semantic analysis of drivable areas and lane lines is necessary for autonomous vehicles to plan reasonable trajectories that comply with traffic rules. Overall, the objective of the perception system is to perform accurate and real-time scene analysis to ensure the safety of autonomous vehicles.

Many works have been done on handling the visual tasks of driving perception. Detection methods such as YOLO (Redmon and Farhadi, 2018; Bochkovskiy et al., 2020) and Faster R-CNN (Ren et al., 2015) can be used for detecting traffic objects in driving scenes. Semantic segmentation algorithms including PSPNet (Zhao et al., 2017) and UNet (Ronneberger et al., 2015) can handle the tasks of recognizing drivable area and lane line in the captured images. These works exhibit excellent performance on corresponding single tasks. However, instead of treating each visual task separately, driving perception systems should be built to handle all visual tasks simultaneously, which can decrease the computation overhead and provide timely feedback on environmental changes. Several multi-task models have been proposed. YOLOP (Wu et al., 2022), HybridNets (Vu et al., 2022), and YOLOPV2 (Han et al., 2022) can perform three visual perception tasks simultaneously using a shared encoder for feature extraction and multiple decoder heads for specific tasks. However, with a primary goal of achieving autonomous driving on complex scenes such as cities or highways, these works design strong perception models at the expense of substantially intensive computation. As a result, they rely on dedicated GPU devices to achieve real-time inference (Miraliev et al., 2023). In contrast, the demand for affordable autonomous driving solutions in simpler environments has been ignored. For instance, in uncomplicated settings such as ports (Qin et al., 2020; Rose et al., 2022), lightweight models with smaller datasets may deliver satisfactory results while minimizing computational requirements and enhancing inference speed. By alleviating the computational demand imposed on deployment hardware, the research on lightweight perception network can potentially pave the way for wider deployment of self-driving. Hence, this work introduces a lightweight multi-task model, thereby contributing to the broader realization of autonomous driving.

We propose a novel multi-task network that can efficiently handle the tasks of traffic object detection, drivable area segmentation, and lane line segmentation simultaneously. The network contains an encoder for feature extraction and two decoders (i.e., detect head and segment head) for object detection and segmentation tasks separately. By adopting MobilenetV2 (Sandler et al., 2018) as the backbone network, the multi-task network has great advantages in the amount of computation and inference speed. For the design of decoders, our implementation of the detect head is based on the YOLOv4 (Bochkovskiy et al., 2020) whose accuracy and speed have been demonstrated on many detection tasks. The segment head is a multi-class segmentation branch that can solve two segmentation tasks altogether. We design a novel loss combination composed of generalized dice loss (Sudre et al., 2017) and focal loss for the multi-class segmentation head. Our network is trained end-to-end on the BDD100K dataset (Yu et al., 2020). It achieves an inference speed of 58 FPS on the Nvidia Tesla V100. The network has also attained competitive performance on all three tasks compared with other multi-task networks. Besides, we carry out ablation experiments to verify the effectiveness of the design of the segment head and the scheme of how the segmentation head obtains features from the encoder.

In summary, the main contributions of this work are: (1) We propose a novel lightweight multi-task network that can simultaneously handle three visual tasks for autonomous driving perception with significantly low computation and high inference speed. (2) Compatible loss functions and data augmentation methods are designed for the proposed network. (3) We validate the performance of the driving perception model and the effectiveness of the multi-task architecture with ablation studies on the BDD100K dataset.

## 2 Related work

The object detection algorithms (Ren and Wang, 2022) can be divided into two categories, namely one-stage methods and two-stage methods. The two-stage object detection models (Girshick et al., 2014; Ren et al., 2015) comprise a region proposal mechanism, in which features in the proposed regions are used to predict the location and the category of objects. The two-stage methods can produce excellent performance, but they are usually slower than the one-stage methods such as YOLO (Redmon et al., 2016). Difference from the two-stage methods that have a network for region proposal, one-stage methods divide the picture into multiple regions with grids for object detection. They subsequently generate multiple proposals in each region and predict the corresponding category of the objects in each proposal. More recently, Yolov4 (Bochkovskiy et al., 2020) and Yolov7 (Jiang et al., 2022; Wang et al., 2023) improved the YOLO architecture by utilizing the advanced model designing tricks, activation functions, and loss functions.

The recognition of drivable area and lane line can be regarded as segment tasks (Zou et al., 2019; Mo et al., 2022; Zhou et al., 2022). Many models based on convolutional neural networks (CNN) have achieved good results in semantic segmentation tasks (Wang et al., 2021). FCN (Long et al., 2015) introduced a full convolutional network to perform semantic segmentation for the first time. It discarded the fully connected layer at the end of VGG and replaced it with a CNN module that can perform dense category prediction, but it is still limited to low-resolution segmentation. U-Net (Ronneberger et al., 2015) uses a U-shaped encoder-decoder structure. The encoder involves convolution and downsampling, and the decoder is composed of upsampling and convolution blocks. PSPNet (Zhao et al., 2017) proposes to extract features in multiple scales through the pyramid pooling module, and then use the merged features for semantic segmentation. Enet (Paszke et al., 2016) improves the inference speed by reducing the size of the feature map.

Multi-task learning is to train models that can perform multiple vision tasks simultaneously. The MultiNet (Teichmann et al., 2018) uses an encoder to extract features that can be used for three decoders to perform scene classification, object detection and driving area segmentation altogether. YOLOP (Wu et al., 2022) uses a similar encoder-decoder architecture. The model is composed of a detection branch based on YOLOv5s for traffic object detection and two simple segment heads for two segmentation tasks. Following YOLOP, Miraliev et al. (2023) compares the inference speed, memory efficiency and energy consumption of the models using the same architecture but composed of three different backbone [i.e., RegNetY (Radosavovic et al., 2020), RexNet (Han et al., 2021), and MobileNetV3 (Howard et al., 2019)]. HybridNets (Vu et al., 2022) chooses EfficientNet (Tan and Le, 2019) as the backbone and uses Bifpn as the neck, which improves the performance on vision tasks but results in reduced inference speed. This model utilizes a multi-class segment head to perform drivable area segmentation and lane line detection altogether.

## 3 Methodology

**Figure 1:**
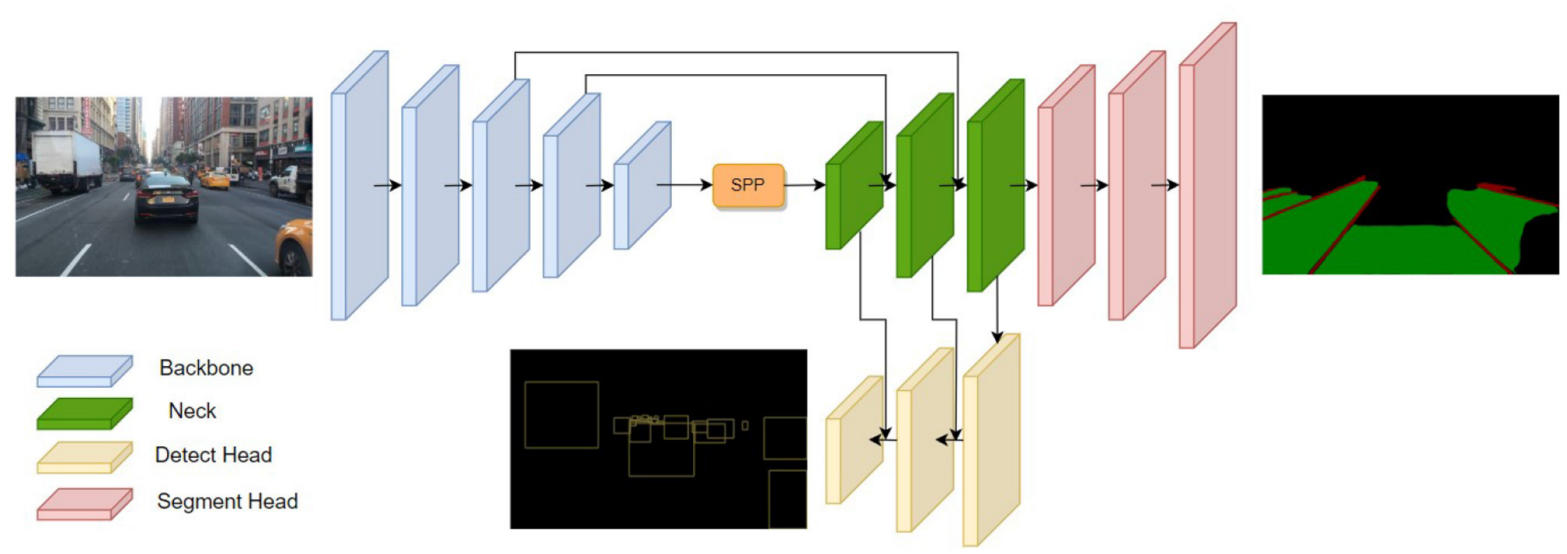
The architecture of Mobip. Mobip contains one shared encoder for feature extraction and two decoders for object detection and semantic segmentation.

We present a lightweight network for multi-task learning of traffic object detection, drivable area segmentation and lane line detection. As shown in Figure 1, our driving perception network, termed Mobip, comprises a shared encoder and two decoders. The feature extracted by the encoder from input images is shared by two decoders, thereby conserving computational resources. Two decoders are designed for handling three visual tasks altogether, specifically a detect head for traffic object detection, and a segment head for drivable area segmentation and lane line detection.

### 3.1 Encoder

The shared encoder comprises two components, i.e., a backbone network and a neck network. The backbone network serves a critical role in the driving perception network as a feature extractor. Contemporary network architectures often leverage networks that exhibit high accuracy on the ImageNet dataset for feature extraction purposes. MobileNetV2 is a lightweight backbone network that has achieved excellent results in both object detection and semantic segmentation tasks (Sandler et al., 2018). By using the inverse residual module as the basic building block, MobileNetV2 achieves low-latency inference in edge devices with fewer parameters and reduced computational complexity. As we aim to design a lightweight driving perception network for real-time inference in edge devices, we choose MobileNetV2 as the backbone network of our model. Moreover, apart from the backbone network, the rest modules of the driving perception network are also implemented with inverted residual as the building block to enhance our model's efficiency.

The features extracted by the backbone are subsequently aggregated by the Neck network. The neck network consists of two modules, including Spatial Pyramid Pooling (SPP) (He et al., 2015) for fusing features of different scales and Feature Pyramid Network (FPN) (Lin et al., 2017a) for fusing features at different semantic layers. Within the FPN module, high-level features are concatenated with low-level features after bilinear interpolation. The neck network allows the features entering the decoders to contain rich semantics information required for the learning of downstream visual tasks.

### 3.2 Decoders

The tasks of driving perception are divided into either a detection task or a segmentation task and subsequently performed by the corresponding task head. Similar to YOLOv4 (Bochkovskiy et al., 2020), our approach employs an anchor-based multi-scale detection scheme. Multiple feature layers in the FPN are passed to the detect head for further feature aggregation. The Path Aggregation Network (PAN) (Liu et al., 2018) is utilized to perform bottom-up feature fusion, enabling better localization feature extraction. The aggregated multi-scale feature from PAN is then used for traffic object detection. Within the multi-scale feature map, every grid is assigned three anchors with varying aspect ratios. The detect head generates predictions for the offset of position, scaled height and width, as well as the corresponding probabilities and confidences for the class prediction.

For the drivable area segmentation and lane line detection, we adopt a multi-class segmentation head to perform two tasks simultaneously. Compared with previous methods that handle these tasks with two separate segmentation heads, our one-branch method can reduce computational redundancy and achieve faster inference speed. The segment head is connected to the bottom layer of FPN and up-samples the feature maps for three times with the bilinear interpolation method. This process restores the feature map to the original image size, subsequently producing probability estimates for three distinct segment categories, namely, background, drivable area, and lane line. The semantic segmentation process identifies the category with the highest probability for each pixel, designating it as the definitive class assignment, and thus generating the segmentation outcome of the two tasks.

### 3.3 Loss function

In our multi-task learning approach, the loss contains two parts for detect head and segment head respectively. The detection loss $L_{det}$ is a weighted sum of classification loss, object loss and bounding box loss, as shown in Equation (1).


(1) $$\begin{array}{l} L_{det}=L_{class}+\alpha_{1}L_{obj}+\alpha_{2}L_{box}, \end{array}$$

where $L_{class}$ is classification loss, $L_{obj}$ is the loss of the confidence of one prediction. $L_{class}$ and $L_{obj}$ are calculated with focal loss (Lin et al., 2017b), which can force the model to learn hard examples. $L_{box}$ is *CIoU* loss (Zheng et al., 2020) which measures the distance of overlap rate, aspect ratio, and scale similarity between predicted results and ground truth.

For the segmentation tasks, since the data for three categories (i.e., background, drivable area and lane line) are imbalance, we design a hybrid loss including focal loss and generalized dice loss (Crum et al., 2006; Sudre et al., 2017) for the training of segment head, as shown in Equation (2). We use generalized dice loss because of its class re-balancing properties.


(2) $$\begin{array}{l} L_{seg}=L_{Dice}+\beta_{1}L_{Focal}, \end{array}$$

(3) $$\begin{array}{l} L_{Dice}=1-2\frac{\sum_{l=1}^{L}w_{l}\sum_{n=1}^{N}g_{ln}p_{ln}}{\sum_{l=1}^{L}w_{l}\sum_{n=1}^{N}g_{ln}+p_{ln}}, \end{array}$$

(4) $$\begin{array}{l} L_{Focal}=-\frac{1}{N}\overset{L}{\underset{l=1}{\sum }}\overset{N}{\underset{n=1}{\sum }}g_{ln}{\left(1-p_{ln}\right)}^{\gamma }\log\left(p_{ln}\right), \end{array}$$

where *l* is the label of segmentation, *g*_*ln*_ and *p*_*ln*_ is the ground truth for pixel *n* being in class *l*, and *p*_*ln*_ is the predicted probability of pixel *n* belonging to class *l*. *w*_*l*_ is the weight assigned to the *l*th label. When $w_{l}=\frac{1}{\sum_{n=1}^{N}g_{ln}}$, which is the inverse volume of a class, the loss of each class in the segmentation result is weighted equally in the $L_{Dice}$ irrespective of the volume of each class.

Finally, the weighted sum of detection loss and segment loss is used for model training, as shown in Equation (5).


(5) $$\begin{array}{l} L_{all}=\gamma_{1}L_{det}+\gamma_{2}L_{seg}, \end{array}$$

The values of α_1_, α_2_, β_1_, γ_1_, andγ_2_ are tuned for the balance of all losses.

## 4 Experiments

### 4.1 Dataset and experimental setting

We use the BDD100K (Yu et al., 2020) dataset to evaluate the performance of the proposed network and compare it with other algorithms. This dataset is publicly available. It includes annotations for object detection, panoptic segmentation, and lane markings, etc., which can support research on a range of driving perception tasks, thus establishing it as a widely recognized benchmark for evaluating perception algorithms for autonomous driving. The dataset contains 100K images in driver perspective view, 70K for training, 10K for validation, and 20K for testing. As the images of the dataset are collected in different scenes, light conditions, and weather conditions, the model trained in the dataset is robust against various disturbances in the environment.

The Adam optimizer is utilized for model training, with the initial learning rate, β_1_ and β_2_ set to 1 × 10^−2^, 0.937 and 0.999, respectively. Cosine annealing and warm-up are applied to adjust the learning rate (Loshchilov and Hutter, 2016) in the training process. In addition to basic data augmentation techniques including mirror, translation, shearing, rotation and photometric distortion, Mosaic and Mixup (Zhang et al., 2017) are also used to improve the performance of both detection and segmentation. We resize the images from 1, 280 × 720 × 3 to 640 × 384 × 3, for the reason of attaining a good trade-off between inference speed and performance (Hou et al., 2019). The model is implemented with PyTorch (Paszke et al., 2019), and experiments are carried out on the NVIDIA Tesla V100. The source code is released at https://github.com/yeminghui/Mobip.

### 4.2 Results

In this section, we compare Mobip to other representative models on three perception tasks. We first compare Mobip with other multi-tasking methods in terms of parameters, number of computations and inference speed to demonstrate the advantage of lightweight designs. We then evaluate the performance of Mobip on three driving perception tasks, comparing it with both other multi-task methods and networks that focus on a single task.

#### 4.2.1 Model parameter and inference speed

Table 1 presents the comparison of Mobip and two multi-task models (i.e., YOLOP and HybridNets) in terms of parameters, computations and inference speed. By adopting MobileNet as the backbone network and the inverted residual as the building block for the network, Mobip has considerable advantages in the amount of calculation (4.8 G), achieving the fastest inference speed (58 FPS) with the input image size of 640 × 384 × 3 in the TESLA V100 GPU. Notably, the performance comparison between Mobip and HybridNets in each driving perception task is not provided in the following, as the primary focus of HybridNets lies in enhancing perception performance, rather than the lightweight network design and inference speed improvement.

**Table 1:** Computational cost for various multi-task models and the inference speed tested on Nvidia Tesla V100.

**Network**	**Params**	**MACs**	**Speed (fps)**
YOLOP	7.9 M	9.3 G	40
HybridNets	12.8 M	7.8 G	27
Mobip (ours)	7.5 M	4.8 G	58

#### 4.2.2 Traffic object detection result

**Figure 2:**
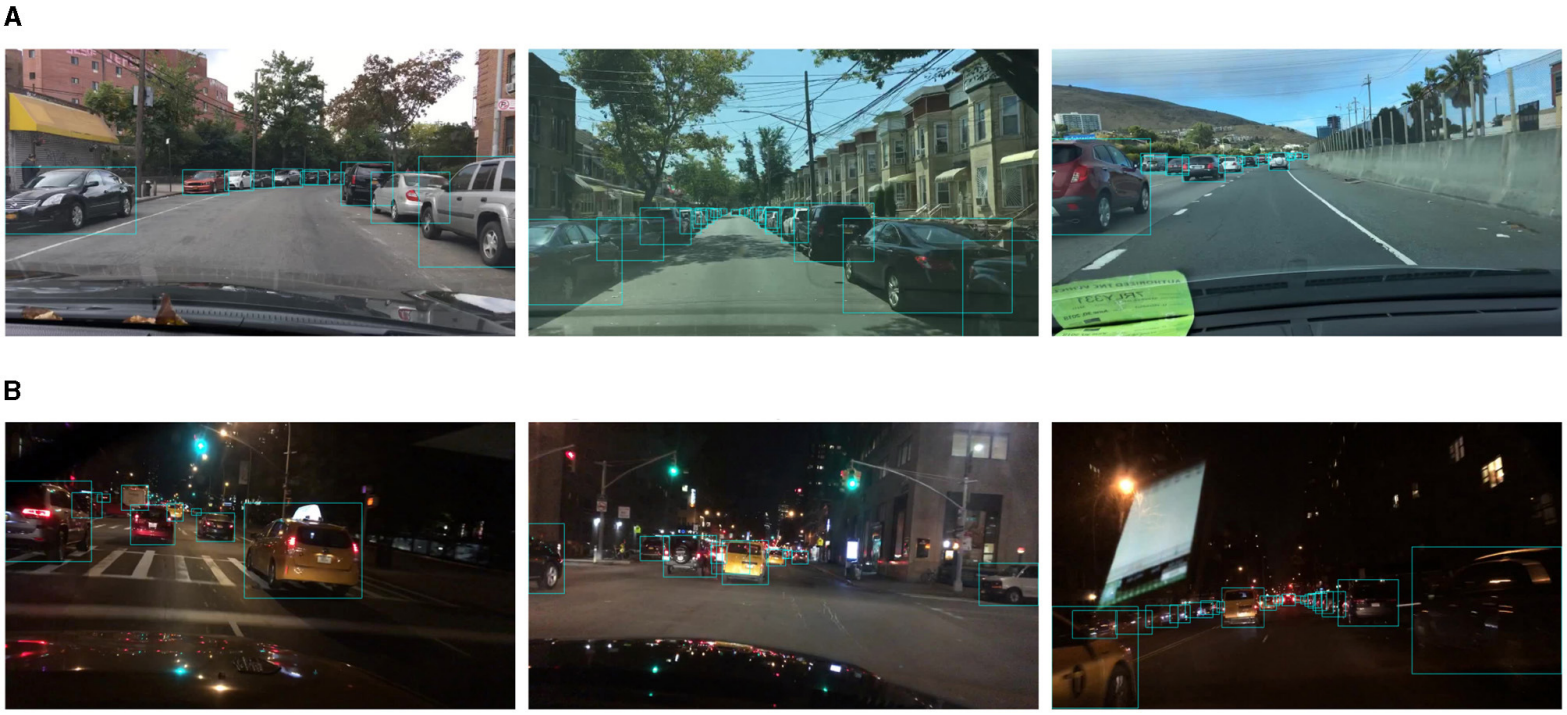
Visualization of traffic object detection results of Mobip. **(A)** Results in day conditions and **(B)** Results in night conditions.

The quantitative comparison in the traffic object detection task is shown in Table 2. We compared Mobip with three multi-task models, including MultiNet, DLT-Net, and YOLOP, and two single-task object detection models, namely Faster R-CNN and YOLOv5s. Mobip achieves the second-best detection result (89.3) on the Recall, higher than YOLOP (89.2) and YOLOv5s (86.8). However, due to the limitations of the MobileNet backbone model, Mobip (75.4) is slightly lower than YOLOP (76.5) and YOLOv5s (77.2) on mAP50. The advantage of Mobip mainly lies in the inference speed. The reason why YOLOv5s achieves higher speed is that it does not perform drivable area segmentation and lane line detection tasks. We visualize the traffic object detection results of Mobip, as shown in Figure 2. Mobip demonstrates excellent performance in both day and night conditions. Surprisingly, it exhibits the capability to detect vehicles that pose challenges for human recognition in low-light environments. Additionally, the model can accurately handle scenes with dense and small objects.

**Table 2:** Results on traffic object detection.

**Network**	**Recall (%)**	**mAP50 (%)**	**Speed (fps)**
Faster R-CNN	81.2	64.9	8.8
YOLOv5s	86.8	77.2	82
MultiNet	81.3	60.2	8.6
DLT-Net	89.4	68.4	9.3
YOLOP	89.2	76.5	40
Mobip (ours)	89.3	75.6	58

#### 4.2.3 Drivable area segmentation result

**Figure 3:**
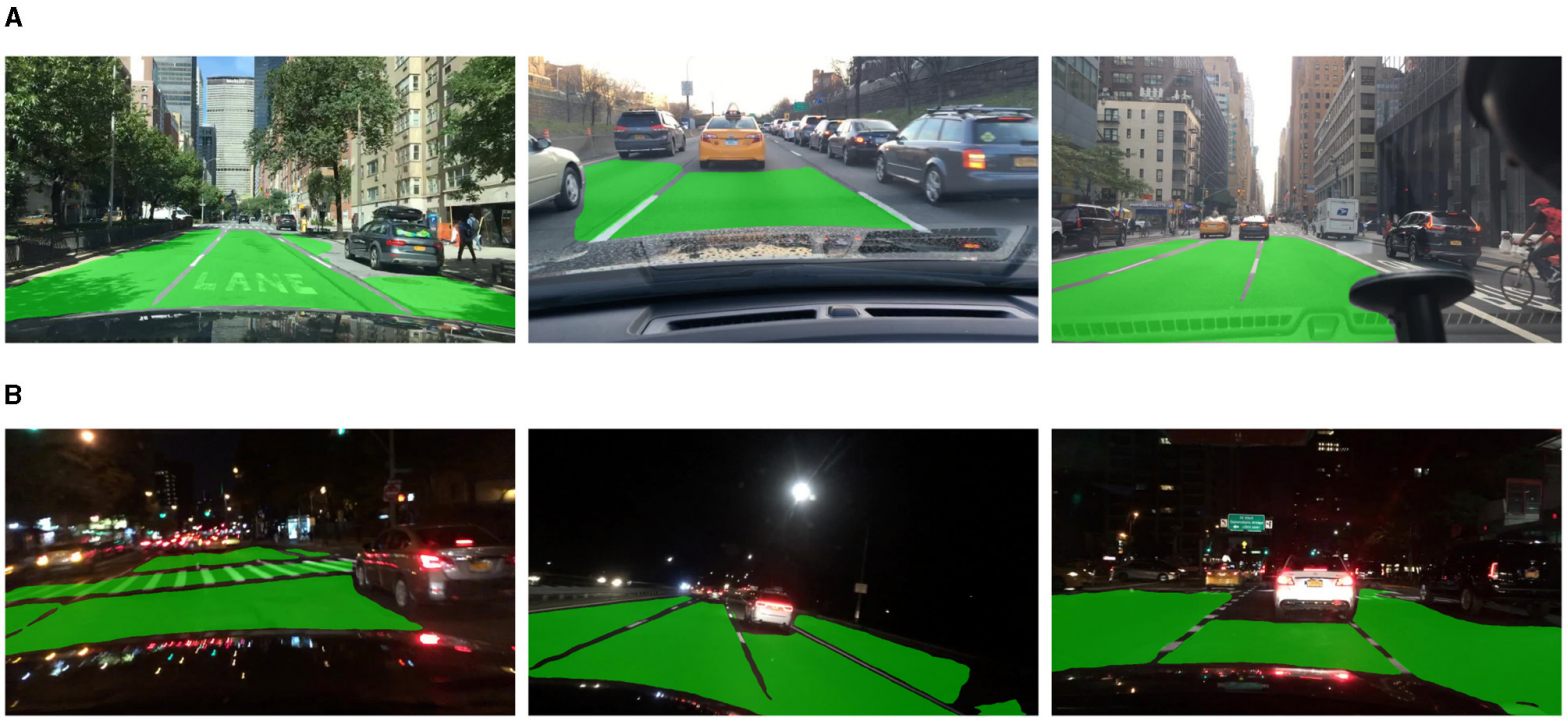
Visualization of drivable area segmentation results of Mobip. **(A)** Day-time result. **(B)** Night-time result.

As shown in Table 3, we compare our model with a single-task segmentation model, PSPNet (Zhao et al., 2017), and three multi-task models including MultiNet, DLT-Net, and YOLOP. Mobip achieved the result of 90.4% on mIoU, outperforming PSPNet in both inference speed and performance. Mobip is working better than all other multi-task networks, apart from YOLOP, the network contains two segment decoders for performing drivable area segmentation and lane line segmentation separately. The performance of Mobip on this task may be limited by the multi-class segmentation branch, which will be verified in the ablation studies. The visualization result of the drivable area segmentation is shown in Figure 3. Mobip performs excellently in this task, demonstrating strong capability in semantic reasoning. The examples show that it can judge whether it is a drivable area based on the cars and lane lines in the surroundings.

**Table 3:** Results on drivable area segmentation.

**Network**	**mIoU (%)**	**Speed (fps)**
PSPNet	89.6	11.1
MultiNet	71.6	8.6
DLT-Net	71.3	9.3
YOLOP	91.5	40
Mobip (ours)	90.4	58

#### 4.2.4 Lane detection result

**Figure 4:**
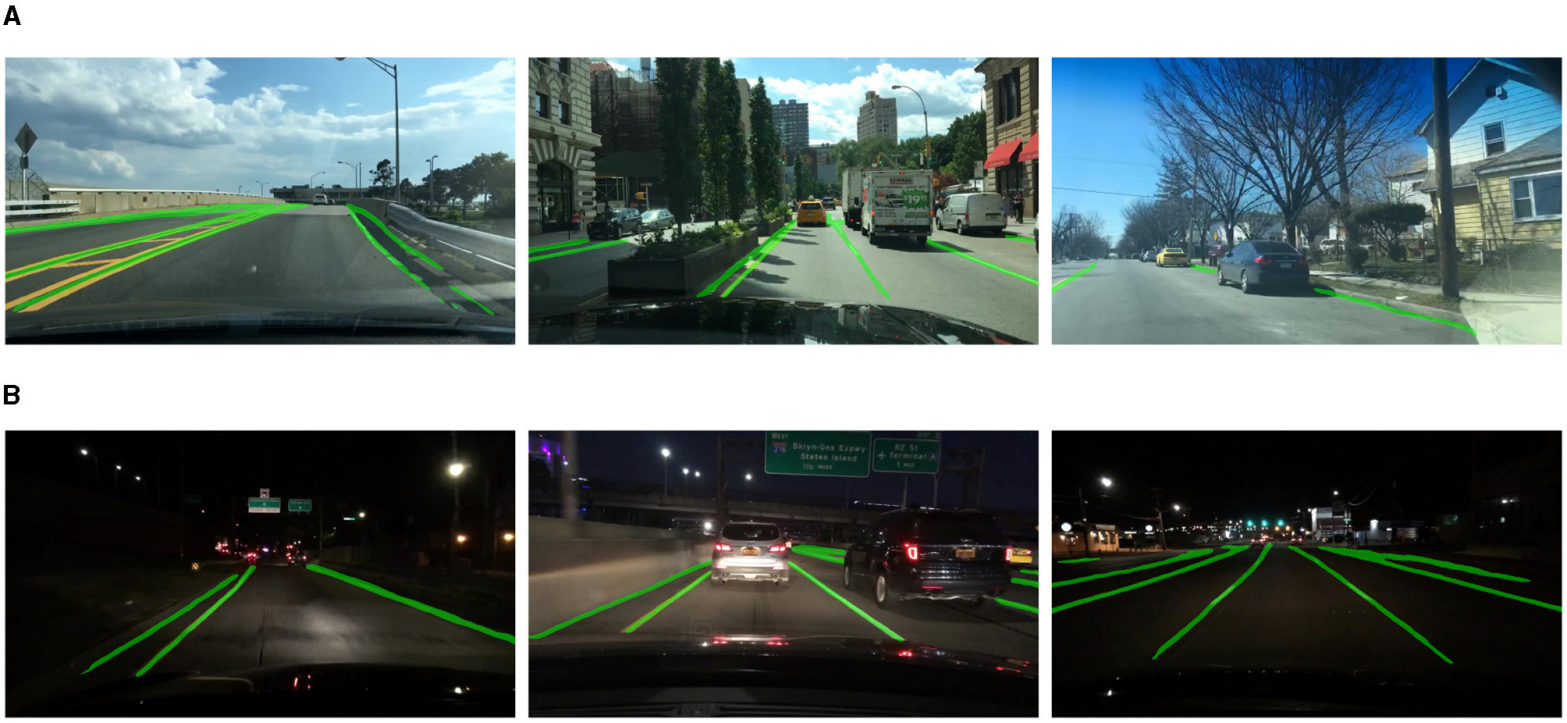
Visualization of lane line segmentation results of Mobip. **(A)** Day-time result. **(B)** Night-time result.

We used IoU and accuracy to evaluate the effectiveness of Mobip in the lane line segmentation task. The result is shown in Table 4. Mobip shows the advantages in performance and speed over other models. In the visualization example as shown in Figure 4, it can be seen that the predicted lane line area is wider than the real lane line, which is consistent with the (30.9%) IoU. Notably, the segment accuracy of Mobip is significantly higher than other networks.

**Table 4:** Results on lane line segmentation.

**Network**	**Accuracy (%)**	**IoU (%)**	**Speed (fps)**
ENet	34.12	14.64	100
SCNN	35.79	15.84	19.8
ENet-SAD	36.56	16.02	50.6
YOLOP	70.5	26.20	40
Mobip (ours)	86.2	30.9	58

### 4.3 Ablation study

We design three ablation studies to verify the effectiveness of the proposed multi-task architecture. The evaluation metrics used on the three tasks are the same as in the last section.

**Figure 5:**
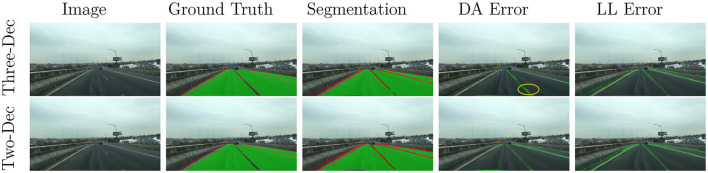
Visualization of the segmentation result of an example image. The data annotations and the segmentation result are presented, in which green areas represent the drivable area and red areas indicate the lane line. Segmentation errors on the drivable area and lane line segmentation tasks are plotted in green areas on the last two columns.

We explore the impact of the design of segment decoders (i.e., one or two segment heads to perform drivable area segmentation and lane line segmentation) on model performance. In previous studies, YOLOP and YOLOPv2 choose the three-decoder architecture which is composed of one detect head and two segment head. They suppose that drivable area segmentation and lane line detection do not share similar features and thus sharing the segment decoder can harm the performance of both tasks. By contrast, similar to the proposed method in this paper, works such as Hybridnet design a multi-category segment head for the recognition of drivable areas and lane lines. These two architectures have not been fairly compared in previous studies because different backbone networks, neck networks and data augmentation methods are used in their works. Therefore, in order to design lightweight and yet effective multi-task networks, we carry out ablation experiments to evaluate the effect of these two architectures. In our experiments, experimental conditions, including data preprocessing methods, data augmentation, parameters of the optimizer, etc., are the same. In the architecture with three decoders, we follow the loss function designed in YOLOP, which is to use cross-entropy loss in the drivable area segmentation task and the weighted sum of cross-entropy and IOU loss in lane line segmentation. In the architecture with two decoders, we adopt the network design proposed in this paper. Table 5 shows the comparison of the performance of these two schemes on all tasks. The architecture with two decoders performs better on traffic object detection compared with the network with three decoders. In the segmentation task, according to the evaluation metrics, the two-decoder architecture exhibits superior performance on the lane line segmentation task, but not as good as the three-decoder architecture on the drivable area segmentation task. To understand the reasons why two-decoder architecture shows poor value in *mIoU* for the drivable area segmentation task, we visualize the segmentation result and the annotations of an example image chosen from the validation set, as shown in Figure 5. By plotting the error map of the drivable area segmentation, we find that the error of the three-decoder scheme is mainly located in the lane line areas. For the two-decoder architecture, the reason for the incorrect segmentation of the drivable area is that the network is conservative on lane line segmentation, such that areas larger than the lane lines are classified as this category. Since there is one class per pixel in this method, the lane line takes up part of the drivable area indicated on the annotation, resulting in lower *mIoU* value in the drivable area segmentation task. Therefore, we suppose that the performance of these two architectures is similar on drivable area segmentation, even though it is not reflected on *mIoU*. Moreover, the advantage of inference speed demonstrates the computational efficiency of the proposed multi-task network.

**Table 5:** Performance comparison of two multi-task frameworks, specifically a three-decoder architecture with one detect head and two segment heads for recognizing drivable area and lane line separately, and a two-decoder network with one detect head and one multi-class segment head to perform two segmentation tasks collectively.

**Multi-task framework**	**Recall (%)**	**AP**	**mIoU**	**Accuracy**	**IoU**	**Speed (fps)**
Three decoders	88.9	74.4	92.6	75.4	30.7	54
Two decoders	89.3	75.6	90.4	86.2	30.9	58

We have also studied the schemes for connecting the segment head to the encoder. While it is clear that semantic layers at all scales in FPN are important for detection, it is still unknown which feature layer should be used to complete the segmentation tasks. We compared two types of features extracted from FPN for segmentation, specifically features of the bottom layer (i.e., the layer with largest scale) of FPN and the merged feature from layers at all scales in FPN. For the methods of mixing feathers at different scales of FPN, our implementation is to add all feature layers together after resizing them to the dimension of the bottom layer of FPN. The result is listed in Table 6. It can be seen that the ways in which the segmentation head obtains features from FPN exert no effect on the performance of the detection task, but training the segment head with the merged feature results in slightly improved accuracy in the lane line segmentation task. This implies that features at lower layers are important for the lane line segmentation task. However, the operation of merging feathers greatly harms the inference speed. To achieve higher inference speed, we choose to connect the segment head with the bottom layer of FPN in the proposed method. Besides, to verify the effectiveness of the proposed multi-task framework, we tested the performance of the model under single-task and multi-task training, as shown in Table 7. Compared with single-task training, the performance of multi-task training is slightly reduced in traffic object detection and lane line segmentation. However, with the design of the shared encoder, the multi-task architecture can complete multiple tasks simultaneously with reduced computation, which is an important feature that autonomous driving perception systems value.

**Table 6:** Performance comparison of two schemes for extracting features from FPN to the segment head.

**Feature sharing scheme**	**Recall (%)**	**AP**	**mIoU**	**Accuracy**	**IoU**	**Speed (fps)**
Merged feature	89.3	75.6	90.1	88.9	30.6	47
Bottom layer	89.3	75.6	90.4	86.2	30.9	58

One is mixing features from three semantic layers of FPN. The other one is only using the bottom feature layer of FPN for segmentation.

**Table 7:** Performance of single-task training and multi-task training.

**Training method**	**Recall (%)**	**AP**	**mIoU**	**Accuracy**	**IoU**
Detect (only)	89.7	76.1	–	–	–
Segment (only)	–	–	90.3	87.0	31.2
Multi-task training	89.3	75.6	90.4	86.2	30.9

## 5 Conclusion

In this paper, we put forward a novel multi-task network that can simultaneously perform three driving perception tasks including traffic object detection, drivable area segmentation and lane detection. Compared with other multi-task models, our model shows advantages in the inference speed while still delivering similar or superior performance on all three driving perception tasks when trained on the BDD100K dataset. Moreover, the effect of several multi-task architecture designs has been validated. Specifically, recognizing drivable area and lane line with a multi-class segment head is superior to handling these two tasks with two segment heads separately. The feature extraction scheme of connecting the segment head to the bottom layer of FPN has also been verified to be efficient. Overall, the proposed network, Mobip, is extremely lightweight yet effective on all three visual tasks.

However, although using MobileNet as the backbone brings advantages in inference speed, it comes with the price of performance limitations. Moreover, the design of using a single segmentation head to perform multi-class segmentation may not be able to generalize to all cases, especially when one segment class is a subset of another one. The proposed multi-task network is also limited to performing three tasks at the same time. In the future, we will introduce other kinds of tasks to the model, such as traffic light recognition.

## Data availability statement

The original contributions presented in the study are included in the article/supplementary material, further inquiries can be directed to the corresponding author.

## Author contributions

MY: Conceptualization, Formal analysis, Methodology, Validation, Visualization, Writing—original draft. JZ: Funding acquisition, Project administration, Resources, Supervision, Writing—review & editing.
